# Aspartic acid supplementation ameliorates symptoms of diabetic kidney disease in mice

**DOI:** 10.1002/2211-5463.12862

**Published:** 2020-05-18

**Authors:** Saki Ichikawa, Tomohito Gohda, Maki Murakoshi, Zi Li, Eri Adachi, Takeo Koshida, Yusuke Suzuki

**Affiliations:** ^1^ Department of Nephrology Juntendo University Faculty of Medicine Bunkyo‐ku Tokyo Japan

**Keywords:** aspartic acid, diabetic kidney disease, metabolomics, nitric oxide

## Abstract

Diabetic kidney disease (DKD) is among the most common and serious complications of both type 1 and type 2 diabetes. In this study, we used KK/Ta‐*Ins*2*^Akita^* (KK‐Akita) mice as a model of DKD and KK/Ta (KK) mice as controls to identify novel factors related to the development/progression of DKD. Capillary electrophoresis coupled with mass spectrometry analysis revealed that circulating Asp (l‐aspartic acid) levels in diabetic KK‐Akita mice tend to be lower than those in control KK mice. Therefore, we evaluated the effect of Asp supplementation to prevent the progression of DKD in KK‐Akita mice. Mice were divided into three groups: (a) untreated KK mice (Control group), (b) untreated KK‐Akita mice (DKD group), and (c) treated (double‐volume Asp diet) KK‐Akita mice (Tx group). Kidney sections were stained with fluorescein isothiocyanate‐labeled lectins, wheat germ agglutinin (WGA), and anti‐endothelial nitric oxide synthase (eNOS) antibody for evaluation of endothelial surface layer (ESL) and NO synthesis. The mesangial area and glomerular size in the DKD group were significantly larger than those in the Control group; however, there was no significant difference in those between the DKD and Tx groups. Albuminuria, the ratio of foot process effacement, and thickness of glomerular basement membrane in the Tx group were significantly lower than those in the DKD group. Furthermore, the expression levels of glomerular WGA and microvascular eNOS in the Tx group improved significantly and approached the level in the Control group. In conclusion, the improvement of albuminuria in the Tx group may be caused by the reduction of oxidative stress in the kidneys, which may lead to the subsequent improvement of glomerular ESL.

## Graphical abstract

Serum aspartic acid (Asp) levels in KK‐Akita mice were lower than those in KK mice. Albuminuria was decreased, and the expression of glomerular wheat germ agglutinin and microvascular endothelial nitric oxide synthase was improved after Asp supplementation in KK‐Akita mice. These results may be caused by the reduction of oxidative stress in the kidneys, which may lead to the subsequent improvement of glomerular endothelial surface layer.

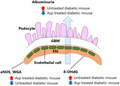

**Abbreviations**
8‐OHdG: 8‐hydroxy‐2′‐deoxyguanosine
Arg: l‐arginine
Asp: l‐aspartic acid
BW: body weight
CE‐MS: capillary electrophoresis coupled with mass spectrometry
Citrulline: l‐citrulline
DKD: diabetic kidney disease
eNOS: endothelial nitric oxide synthase
ESL: endothelial surface layer
FBG: fasting blood glucose
FITC: fluorescein isothiocyanate
GBM: glomerular basement membrane
GD: glomerular diameter
KK: KK/Ta
KK‐Akita: KK/Ta‐Ins2Akita
NO: nitric oxide
PAS: periodic acid–Schiff
PLS‐DA: projection on latent structure discriminant analysis
VIP: variable importance in the projection
WGA: wheat germ agglutinin

Diabetic kidney disease (DKD) is among the most common and serious complications of both type 1 and type 2 diabetes; it is heterogeneous in terms of natural history, pathogenesis, and treatment response [1]. The prevalence of DKD has remained fairly stable over the last two decades despite advances in the treatment of hyperglycemia, hypertension, and dyslipidemias, as well as the widespread use of renin–angiotensin–aldosterone system inhibitors [2, 3, 4]. These therapies aim to slow the progression of DKD; however, eventually many patients with diabetes reach end‐stage renal disease, which requires renal replacement therapy [5]. The complex mechanisms leading to the development and progression of renal injury in DKD are not well understood; however, current knowledge indicates that the pathogenesis of DKD is multifactorial, where inflammation and oxidative stress may be the relevant factors [5, 6].

Metabolomics is an approach of systems biology that provides global metabolic information regarding biological samples [7]. This approach has been widely applied to further understand the pathogenesis of many diseases. Metabolomics can also be applied to establish diagnoses and select the most appropriate treatments [8, 9, 10]. In the present study, we performed capillary electrophoresis coupled with mass spectrometry (CE‐MS) analysis using the sera of diabetic mice to identify novel factors related to the development/progression of DKD. For this purpose, we chose KK/Ta‐*Ins*2*^Akita^* (KK‐Akita) mice as a model of DKD and KK/Ta (KK) mice as controls. KK‐Akita mice exhibit diabetes‐related phenotypes such as impaired levels of fasting blood glucose (FBG), hemoglobin A1c, and albuminuria [11]. Significant changes of amino acid profile in urea cycle were observed between KK‐Akita and KK mice. In particular, we focused on l‐aspartic acid (Asp), one of the nonessential amino acids. Circulating Asp levels in KK‐Akita mice are decreased compared with those in KK mice at 6 weeks of age, and this decrease can persist even in 15‐week‐old mice. Additionally, Asp is required for the conversion of l‐citrulline (Citrulline) to l‐arginine (Arg) [12]. Arg is a substrate for nitric oxide (NO). Endothelial dysfunction, which is characterized by the reduced bioavailability of NO, plays an important role in the pathogenesis of vascular complications in diabetes [13]. Reduced NO production or increased NO degradation may contribute to kidney dysfunction in different pathological conditions, including diabetes [14]. Although several studies demonstrated the usefulness of Arg and/or Citrulline supplementation to prevent progression of DKD [14, 15], whether Asp supplementation is beneficial for patients with DKD remains unknown. Thus, this study aimed to evaluate the effect of Asp supplementation in preventing the progression of DKD in diabetic KK‐Akita mice.

## Methods

### Mice

All animal experiments were approved by the Animal Committee at Juntendo University Faculty of Medicine, and all animals were treated according to the guidelines for animal experimentation at Juntendo University (Permit Number: 310037). All surgery and sacrifice were performed under sodium pentobarbital anesthesia, and all efforts were made to minimize suffering.

All mice were individually housed in plastic cages with free access to food (CE‐2; CLEA Japan, Tokyo, Japan) and water throughout the experimental period, maintained in the same room under conventional conditions with a regular 12‐h light/dark cycle, and a controlled temperature at 24 ± 1 °C.

Male KK‐Akita mice were inherited by Akita University (Akita, Japan) (H. Fujita, from Akita University School of Medicine, kindly provided the male KK‐Akita mice). KK‐Akita mice were generated by backcrossing male C57BL/6‐Akita mice to female KK mice for 10 generations at Akita University [11]. We purchased female KK mice (CLEA Japan) and mated them with male KK‐Akita mice to keep the strain. Genotyping was performed as described previously [16].

Mice were divided into three groups: (a) untreated KK mice (Control group; *n* = 5), (b) untreated KK‐Akita mice (DKD group; *n* = 5), and (c) treated (fed a specified diet, i.e., CE‐2 with doubled Asp) KK‐Akita mice (Tx group; *n* = 5). As shown in Table 1, we prepared a specified diet with a double volume of not only Asp but also asparagine, the precursor of Asp, as reported previously [17]. All groups were weaned by 4 weeks of age and fed CE‐2 until 6 weeks of age. The Control and DKD groups were fed normal chow consistently, and the Tx group was fed CE‐2 with doubled Asp between 6 and 15 weeks of age.

**Table 1:** Component composition of control (CE‐2) and specified diet

	Control diet (CE‐2)	Specified diet
Amino acid (g·100 g^−1^)		
Aspartic acid	2.34	4.68
Asparagine	0.00	2.46
Threonine	1.01	0.96
Serine	1.28	1.22
Glutamic acid	4.35	4.14
Glycine	1.53	1.45
Alanine	1.38	1.31
Valine	1.14	1.08
Isoleucine	0.89	0.85
Leucine	1.80	1.71
Tyrosine	0.81	0.77
Phenylalanine	1.21	1.15
Lysine	1.44	1.37
Histidine	0.59	0.56
Arginine	1.69	1.61
Proline	1.44	1.37
Cystine	0.39	0.37
Methionine	0.44	0.42
Tryptophan	0.24	0.23
Protein (%)	25.33	29.00
Fat (%)	4.38	4.16
Nitrogen‐free extract (%)	49.67	47.23
Water (%)	8.91	8.47
Fiber (%)	4.88	4.64
Ash (%)	6.83	6.49
Energy (kcal)	339.42	342.40

### Biochemical measurements

Urinary albumin excretion, body weight (BW), and FBG levels were measured at 6 and 15 weeks of age. Urine samples were collected daily and were analyzed to detect albuminuria using DCA 2000 microalbumin‐creatinine immunoassay cartridges with a DCA Vantage Analyzer (Siemens Healthcare, Erlangen, Germany). Urinary creatinine was measured using the QuantiChrom Creatinine Assay Kit (BioAssay Systems, Hayward, CA, USA). Urinary nitrates + nitrites were determined using a colorimetric kit (Griess Reaction; R&D Systems, Inc., Minneapolis, MN, USA). Glucose levels were measured by Glutest Mint (Sanwa Kagaku Kenkyusho Co., Ltd., Aichi, Japan) in blood samples obtained from the tail. We measured blood glucose after 6 h of daytime fast.

### Renal histology

For light microscopy, sagittal kidney sections were fixed in 20% formalin and embedded in paraffin, and they (1.5 µm thick) were stained with a periodic acid–Schiff (PAS) reagent. The glomerular axial‐sectional area and the mesangial area were scaled quantitatively with a computer‐aided manipulator (KS400; Carl Zeiss Vision, Munich, Germany) by tracing the outer edge of the glomerular capillary. Five glomerular sections were selected at random from each experimental group by scanning from the outer cortex. PAS‐stained areas (mesangial area) in the glomerular cross‐sectional area were regarded as the relative mesangial areas [18]. The glomerular size was measured as glomerular diameter (GD) using imagej 1.51t software (National Institutes of Health, Bethesda, MD, USA; http://rsb.info.nih.gov/ij). Maximum GD was calculated as the mean of the following two measurements: maximal diameter of the glomerulus and the maximal chord perpendicular to the maximal diameter [19].

### Transmission electron microscopy and morphometry

For transmission electron microscopy, kidney sections were fixed with 2.5% glutaraldehyde in 0.1 m phosphate buffer (pH 7.4), followed by postfixation with 1% OsO4 in the same buffer. Fixed specimens were dehydrated with a graded series of ethanol and embedded in Epok 812 (Oken Shoji, Tokyo, Japan). Ultrathin sections were cut and stained with uranyl acetate and lead citrate. Specimens were examined with an HT7700 transmission electron microscope (Hitachi, Tokyo, Japan).

Foot process effacement is evaluated as the proportion of effacement, which is determined as the length of the effacement, to the length of the capillary. We scaled five glomeruli per mouse and five capillaries per glomerulus for three mice in each experimental group using imagej 1.51t software [20]. Glomerular basement membrane (GBM) was also measured using imagej 1.51t software by measured five glomeruli per mouse and three regions of the GBM per glomerulus in three mice in each experimental group.

### Lectin histochemistry

For lectin histochemistry, fluorescein isothiocyanate (FITC)‐labeled lectins and wheat germ agglutinin (WGA) were purchased from EY Laboratories (San Mateo, CA, USA). Paraffin‐embedded sections were deparaffinized, rehydrated, and blocked in carbohydrate‐free blocking solution (Vector Laboratories, Burlingame, CA, USA). The slides were incubated at 4 °C overnight with lectin aliquoted in carbohydrate‐free blocking solution (FITC–WGA, 15 µg·mL^−1^). Washes were performed with PBS [21]. We randomly selected five glomeruli per mouse and measured the luminance of them in each experimental group using KS 400.

### Immunohistochemical staining

Paraffin‐embedded sections were deparaffinized in xylene and rehydrated in 100%, 90%, 80%, 70%, and 50% ethanol. Microwave treatment was done to activate the enzymes. To reduce the background staining, nonspecific binding was blocked by incubating with blocking solution [PBS (pH 7.2) containing 2% bovine serum albumin, 2% fetal bovine serum, and 0.2% fish gelatin, added mouse IgG blocking reagent (Vector Laboratories)]. The sections were then incubated with the primary antibody [purified mouse anti‐endothelial nitric oxide synthase (eNOS)/NOS Type 3, BD Transduction Laboratories, San Jose, NJ, USA, and anti‐8‐hydroxy‐2′‐deoxyguanosine (8‐OHdG) monoclonal antibody, Japan Institute for the Control of Aging, Shizuoka, Japan] diluted 1 : 100 in blocking solution at 4 °C. Peroxidase activity was developed in 3,3‐diaminobenzidine. Finally, Mayer's hematoxylin was added as a counterstain. The secondary antibody was anti‐mouse Envision Plus polymer reagent (DAKO, Santa Clara, CA, USA).

### Capillary electrophoresis coupled with mass spectrometry analysis

Serum metabolites of the Control and DKD groups at 6 and 15 weeks of age were extracted and analyzed by the Human Metabolome Technologies (HMT Inc., Yamagata, Japan) method. Briefly, methanol containing Internal Standard Solution 1 (HMT Inc.) was added to the sera and mixed well. Subsequently, 200 µL of Milli‐Q water (Merck Millipore, Burlington, MA, USA) and 500 µL of chloroform were added and centrifuged at 2300 ***g*** for 10 min at 4 °C. The upper aqueous layer was collected and then filtrated with a Millipore 5‐kDa cutoff filter and dried. The metabolites were resuspended in 25 µL of Milli‐Q water containing Internal Standard Solution 3 (HMT Inc) and applied to CE‐MS. All CE‐MS experiments were performed using an Agilent 7100 CE capillary electrophoresis system (Agilent Technologies, Santa Clara, Waldbronn, Germany) connected to an Agilent 6530 accurate Q‐TOF‐MS system (Agilent Technologies Inc., Santa Clara, CA, USA).

The data obtained by CE‐MS analysis were preprocessed using masshunter Workstation Software Qualitative Analysis version B.06.00 (Agilent Technologies Inc.). Each metabolite was identified and quantified based on the peak information including *m/z*, migration time, and peak area. The metabolite peaks were analyzed statistically using the mass profiler professional software (Agilent Technologies Inc.).

### Pattern recognition and statistical analysis

*t*‐Test and partial least‐squares projection on latent structure discriminant analysis (PLS‐DA) were carried out using the software metaboanalyst version 4.0 (https://www.metaboanalyst.ca/faces/home.xhtml). To further select differential variables in the PLS‐DA models, the cutoff value of 1.0 was set in the variable importance in the projection (VIP). The selected variables between the Control and DKD groups were then conducted using one‐way analysis of variance with Tukey's honestly significant difference analysis as mounted in ibm spss statistics version 23 software, and a *P* < 0.05 was regarded as statistically significant [22].

## Results

### Blood normalized Asp levels tend to be lower in the DKD mice than in the Control mice at 6 and 15 weeks of age by CE‐MS analysis

**Fig. 1:**
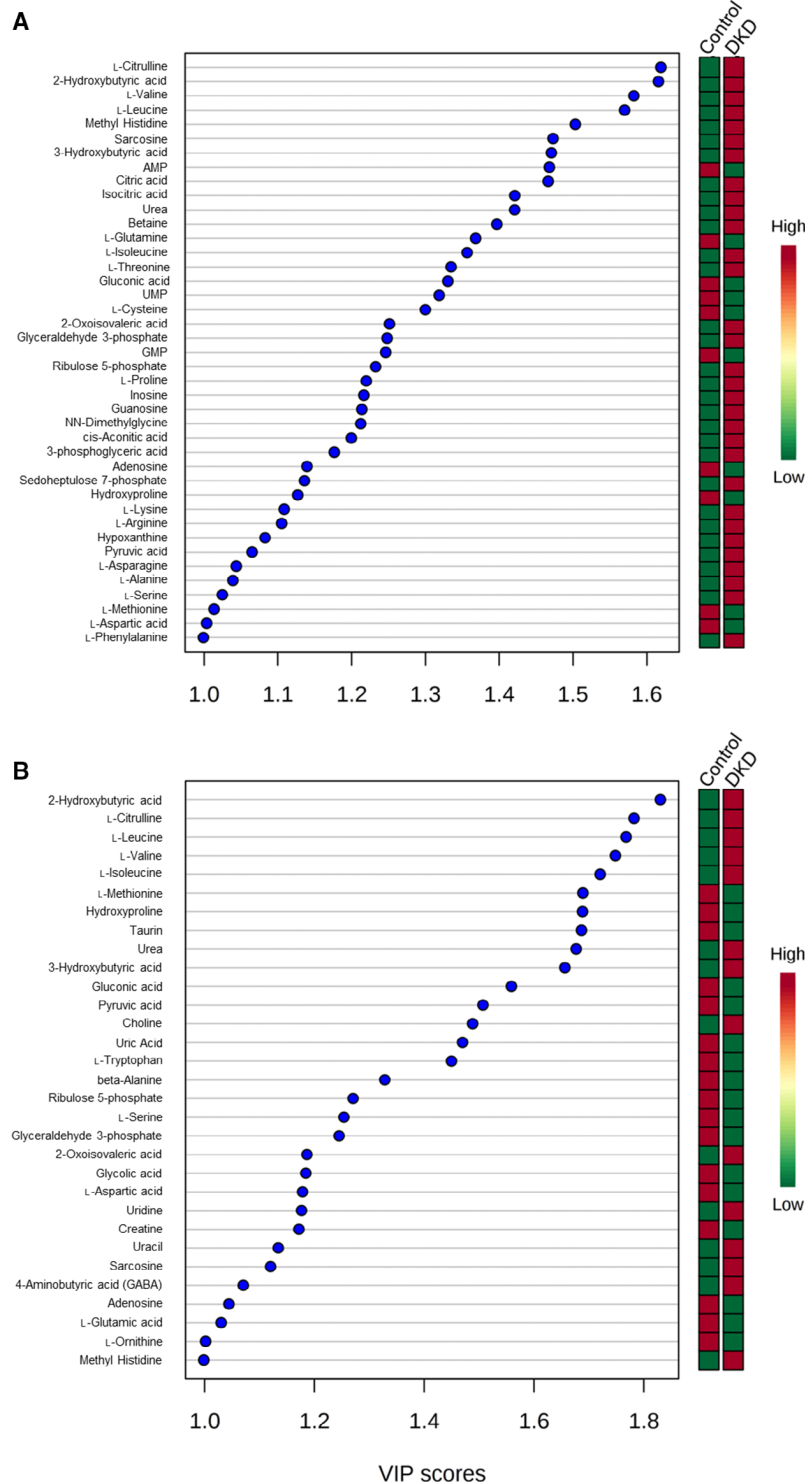
The VIP scores. Multivariate data analysis was conducted with the software metaboanalyst version 4.0. Partial least‐squares PLS‐DA was subsequently conducted using the par‐scaling CE‐MS data. In order to further select differential variables in the PLS‐DA models, the cutoff value of 1 was set in the VIP. Representative VIP scores > 1.0 between the Control and DKD groups at 6 weeks of age (A) and at 15 weeks of age (B).

To sort out the prospective metabolites that specifically changed, we calculated the VIP values from the PLS‐DA models. PLS‐DA models indicated the influence of the metabolites on the classification, and VIP values > 1.0 signified that the changed metabolites contributed significantly to the clustering [22]. Under the criteria of VIP > 1.0 (Fig. 1) and *P* < 0.05, the metabolites shown in Table 2 were identified as potential candidates differentiating the DKD group from the Control group when we compared them at the same weeks of age. Strong or moderate correlations were identified between the metabolites and albuminuria, FBG, and hemoglobin A1c (data not shown). We paid special attention to three metabolites interacting with urea cycle, urea, Citrulline, and Asp.

**Table 2:** The metabolites with *P* < 0.05 and VIP score > 1.0 when comparing KK and KK‐Akita mice at the same weeks of age. UAE, urinary albumin excretion; HbA1c, hemoglobin A1c; GABA, γ‐aminobutyric acid.

	Control versus DKD				Correlation coefficient^a^		
	15 weeks		6 weeks				
	*P*‐value	VIP	*P*‐value	VIP	UAE	FBG	HbA1c
2‐Hydroxybutyric acid	3.87E‐16	1.79	6.80E‐20	1.57	0.97	0.98	0.97
l‐Citrulline	1.40E‐13	1.75	2.09E‐20	1.58	0.94	0.95	0.94
l‐Leucine	5.45E‐13	1.73	2.10E‐15	1.51	0.99	0.93	0.93
l‐Valine	2.78E‐12	1.71	2.49E‐16	1.53	0.98	0.92	0.91
l‐Isoleucine	2.08E‐11	1.68	3.22E‐08	1.31	0.98	0.92	0.91
l‐Methionine	1.47E‐10	1.65	3.28E‐04	1.11	−0.91	−0.89	−0.89
Hydroxyproline	1.53E‐10	1.65	3.55E‐05	1.12	−0.92	−0.89	−0.89
Taurine	1.70E‐10	1.65	–	–	–	–	–
Urea	2.91E‐10	1.65	1.41E‐09	1.37	0.88	0.91	0.90
3‐Hydroxybutyric acid	8.51E‐10	1.62	6.20E‐11	1.42	0.95	0.88	0.86
Gluconic acid	5.04E‐08	1.53	9.29E‐08	1.36	−0.86	−0.82	−0.82
Pyruvic acid	2.76E‐07	1.48	1.28E‐04	1.03	−0.88	−0.79	−0.80
Choline	4.84E‐07	1.46	–	–	–	–	–
Uric Acid	8.12E‐07	1.44	–	–	–	–	–
l‐Tryptophan	1.40E‐06	1.45	–	–	–	–	–
beta‐Alanine	2.27E‐05	1.30	–	–	–	–	–
Ribulose 5‐phosphate	6.76E‐05	1.24	2.46E‐06	1.19	−0.63	−0.68	−0.66
l‐Serine	9.08E‐05	1.24	2.70E‐04	1.01	0.79	−0.67	−0.67
Glyceraldehyde 3‐phosphate	1.06E‐04	1.22	1.55E‐06	1.20	−0.61	−0.66	−0.64
2‐Oxoisovaleric acid	2.72E‐04	1.17	1.41E‐06	1.21	0.68	0.61	0.62
Glycolic acid	2.80E‐04	1.21	–	–	–	–	–
l‐Aspartic acid	3.07E‐04	1.15	3.90E‐04	1.04	−0.56	−0.63	−0.61
Uridine	3.16E‐04	1.16	–	–	–	–	–
Creatine	3.39E‐04	1.16	–	–	–	–	–
Uracil	5.84E‐04	1.15	–	–	–	–	–
Sarcosine	7.09E‐04	1.12	5.27E‐11	1.43	0.59	0.66	0.64
GABA	1.36E‐03	1.06	–	–	–	–	–
Adenosine	1.88E‐03	1.04	2.68E‐05	1.11	−0.51	−0.54	−0.54
l‐Glutamic acid	2.22E‐03	1.02	–	–	–	–	–
Methyl histidine	3.20E‐03	1.01	4.79E‐12	1.45	0.44	0.58	0.58

^a^ Correlation coefficient: correlation coefficient of metabolites with *P* < 0.05 and VIP > 1.0 at 6 and 15 weeks of age in both experimental groups and their phenotype.

**Fig. 2:**
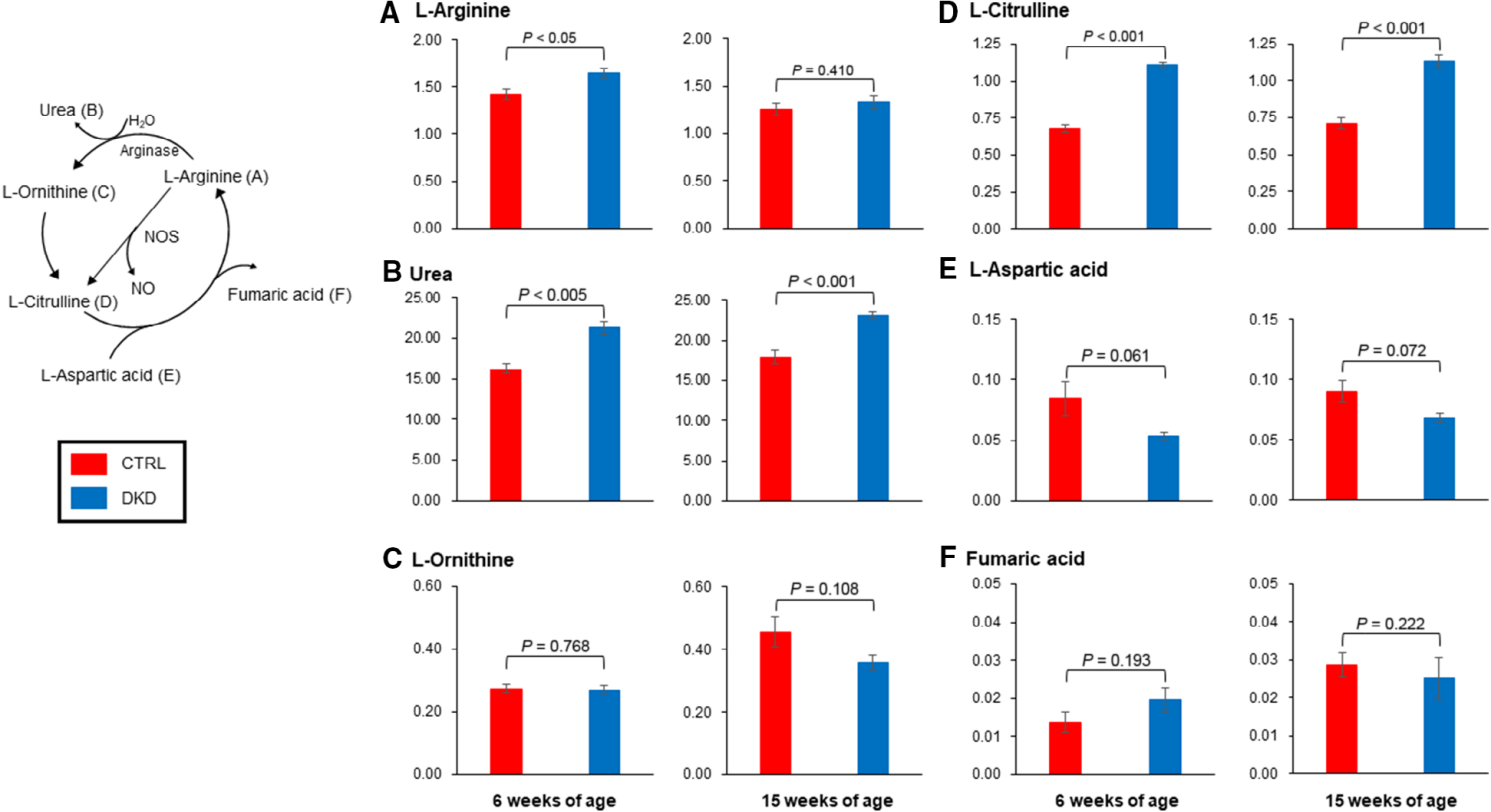
The results of CE‐MS analysis of the amino acids of the urea cycle. The *Y*‐axis shows the relative concentration of each amino acid. The error bars represent SD. The relative concentration of urea and l‐Citrulline in the DKD group (*n* = 5) was significantly higher than that in the Control group (*n* = 5). The relative concentration of l‐aspartic acid in the DKD group tended to be lower than that in the Control group, but no statistical difference was observed.

Figure 2 shows the results of CE‐MS analysis of the metabolites of the urea cycle. The graphs show the normalized concentration of each metabolite in the sera. The results indicate that the levels of urea and Citrulline in the DKD group were significantly higher than those in the Control group when these were compared at the same weeks of age. Additionally, Asp level in the DKD group tended to be lower than that in the Control group, but no statistical difference was observed.

### Blood Asp levels are comparable between the DKD and Control mice after Asp supplementation

Table 3 shows the results of the amino acid analysis in each group at 15 weeks of age. The circulating Asp levels in the DKD group were significantly lower than those in the Control group. However, there was no significant difference in the circulating Asp levels between the DKD and Tx groups (*P* = 0.07). Conversely, the circulating Asp levels in the Tx group were comparable to those in the Control group (*P* = 0.53). The circulating levels of Arg and Ornithine did not differ among three groups. The circulating citrulline levels, which were significantly higher in the DKD group than in the Control group, did not change after the Asp supplementation. The ratio of Arg to citrulline in the DKD group was significantly lower than that in the Control group. Again, the ratio of Arg to Citrulline was not significantly different between the DKD and Tx groups.

**Table 3:** Amino acid analysis in each mouse at 15 weeks of age. Data expressed as means ± standard deviation.

Amino acid	Control	DKD	Tx
l‐Aspartic acid (nmol)	0.23 ± 0.04	0.16 ± 0.03^a^	0.32 ± 0.24
l‐Arginine (nmol)	2.03 ± 0.16	1.83 ± 0.18	1.90 ± 0.23
l‐Ornithine (nmol)	1.24 ± 0.24	1.08 ± 0.11	0.95 ± 0.25
l‐Citrulline (nmol)	1.86 ± 0.14	2.24 ± 0.09^a^	2.32 ± 0.14^c^
l‐Arginine/l‐Citrulline ratio	1.17 ± 0.08	0.87 ± 0.07^b^	0.84 ± 0.05^c^

^a^ *P*‐value < 0.05: Control versus DKD at the same weeks of age

^b^ *P*‐value < 0.001: Control versus DKD at the same weeks of age

^c^ *P*‐value < 0.001: Control versus Tx mice at the same weeks of age.

### Albuminuria is significantly decreased after Asp supplementation in the DKD mice

As shown in Table 4, the levels of albuminuria in the DKD group were significantly increased in mice as early as 6 weeks of age compared with those in the Control group, and albuminuria became more pronounced as the mice aged, whereas the levels of albuminuria in the Tx group were significantly lower than those in the DKD group at 15 weeks of age. The BW, which was significantly lower in the DKD group than in the Control group at 6 and 15 weeks of age, did not differ between the DKD and Tx groups. The ratio of the kidney weight to BW at 15 weeks of age was significantly higher in the DKD group than in the Control group; however, the ratio in the Tx group did not change after Asp supplementation. The levels of FBG did not differ between the DKD and Tx group at 6 and 15 weeks of age, although those in the DKD group were significantly higher than those in the Control group.

**Table 4:** Biochemical parameters in each group at 6 and 15 weeks of age. Data expressed as means ± standard deviation, or median (25th percentile, 75th percentile). UAE, urinary albumin excretion.

	Control	DKD	Tx
Number of mice	5	5	5
6 weeks of age			
BW (g)	30.8 ± 0.6	23.8 ± 0.2^b^	23.6 ± 0.9
UAE (µg·day^−1^)	51 (33, 106)	954 (448, 1283)^a^	1091 (1005, 1383)
FBG (mg·dL^−1^)	212 ± 11	592 ± 17^b^	622 ± 15
15 weeks of age			
BW (g)	40.0 ± 0.9	28.8 ± 0.3^b^	26.1 ± 1.0
Kidney weight/BW (%)	0.59 ± 0.03	1.23 ± 0.19^b^	1.11 ± 0.06
UAE (µg·day^−1^)	56 (16, 71)	2219 (1713, 2547)^b^	1470 (1035, 1669)^c^
FBG (mg·dL^−1^)	210 ± 2	691 ± 13^b^	692 ± 10

^a^ *P*‐value < 0.05: Control versus DKD group at the same weeks of age

^b^ *P*‐value < 0.001: Control versus DKD group at the same weeks of age

^c^ *P* ‐value < 0.05: DKD versus Tx group at the same weeks of age.

### Glomerular podocyte and endothelial damage are improved after Asp supplementation in the DKD mice

**Fig. 3:**
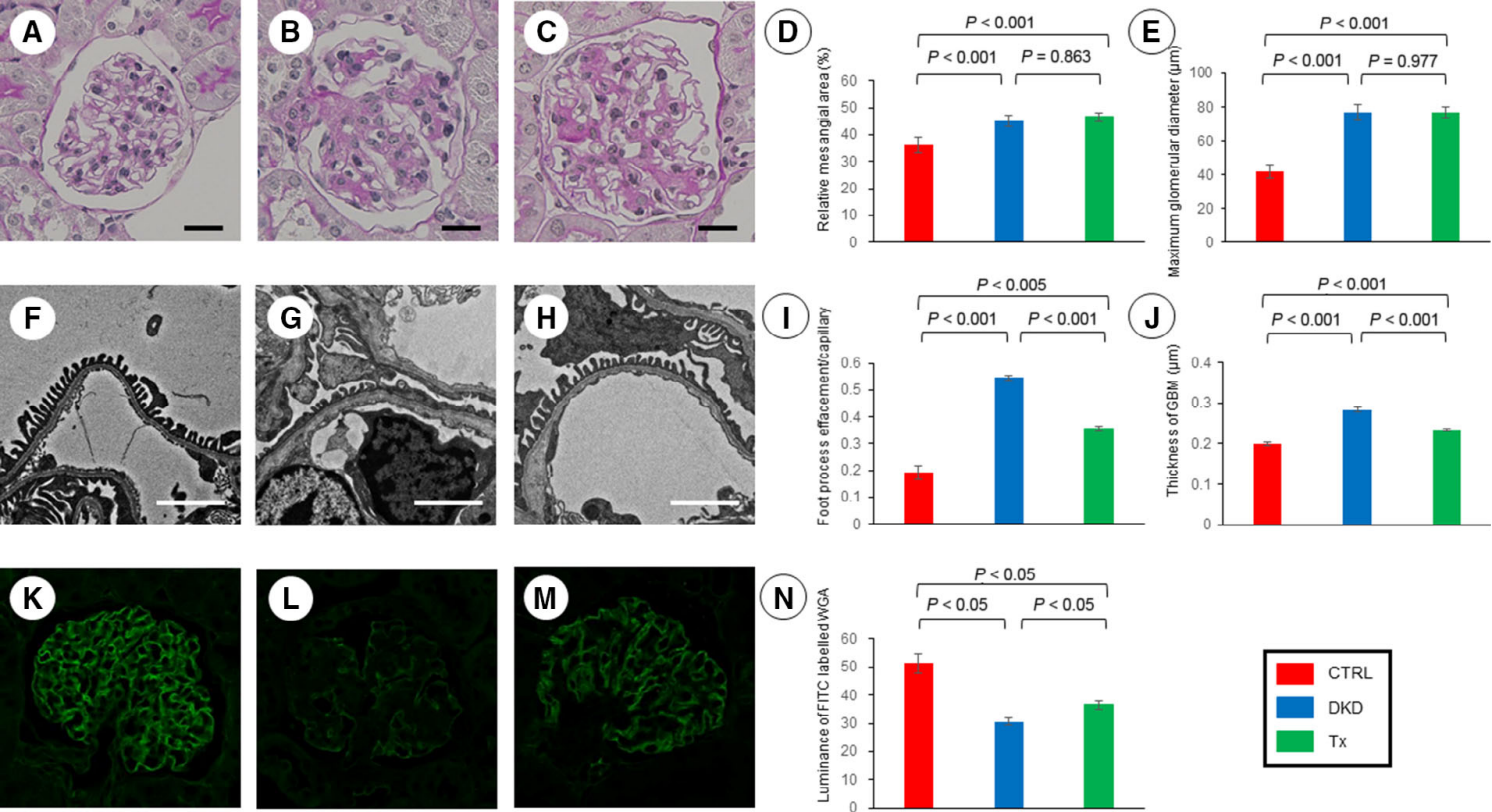
The renal pathology in each group. The mesangial proliferation and foot process effacement were more progressive in both of the DKD and Tx groups compared with the Control group. The mesangial proliferation level and the glomerular size did not differ between the DKD and Tx groups, whereas the rate of foot process effacement per capillary and the GBM thickness were decreased in the Tx group compared with the DKD group. Furthermore, the glomerular ESL shown by WGA staining level in the Tx group was significantly higher than the DKD group. Representative kidney sections of periodic acid–Schiff staining (400‐fold magnification) in the Control group (A), DKD group (B), and Tx group (C). The length of each scale bar shows 10 µm. Quantitative analysis of the mesangial area (D) and the glomerular size (E) (*n* = 5 per group, 5 glomeruli randomly selected in each mouse). Representative kidney sections of electron micrographs (2000‐fold magnification) in the Control group (F), DKD group (G), and Tx group (H). The length of each scale bar shows 2 µm. Quantitative analysis of foot process effacement (I) (*n* = 3 per group, 5 glomeruli, and capillaries per mouse) and the thickness of GBM (J) (*n* = 3 per groups, 5 glomeruli, and 3 regions of the GBM per glomerulus). WGA staining for ESL in glomeruli in the Control group (K), DKD group (L), and Tx group (M). Quantitative analysis of the luminance of FITC (*n* = 5 per group, 5 glomeruli randomly selected in each mouse) (N). The error bars represent SD (D, E, I, J, and N).

Renal histopathology was assessed by light and electron microscopy at 15 weeks of age. Figure 3A–C shows the representative glomerular light micrographs from each group. Remarkable mesangial expansion, as evidenced by increased accumulation of PAS‐positive material in the mesangial area, was observed in the DKD group, whereas mesangial expansion was relatively mild in the Control group. Quantitative analysis of PAS‐stained glomerular sections by KS400 did not differ between the DKD group and the Tx group (Fig. 3D). The pattern of changes in glomerular size, estimated by Max GD, among the three groups was similar to that of mesangial expansion (Fig. 3E). Figure 3F–H shows the representative glomerular electron micrographs from each group. Morphometric analysis revealed a significant increase in the rates of foot process effacement and GBM thickness in the DKD group compared with the Control group. Additionally, the rates of foot process effacement and GBM thickness were significantly lower in the Tx group compared with the DKD group (Fig. 3I,J).

The glomerular endothelial surface layer (ESL) was detected by lectin staining (Fig. 3K–M). Paraffin‐embedded specimens were stained with WGA lectin, which binds to sugar residues, sialic acid, and *N*‐acetylglucosaminyl residues of glycoproteins. WGA lectin was observed on the glomerular loop in each group. The disarray of WGA staining was demonstrated in the DKD group but was significantly ameliorated by treatment with Asp in the Tx group. Quantitative analysis of FITC luminance in the glomerular sections by KS400 revealed a significantly greater improvement in the Tx group compared with the DKD group (Fig. 3N).

**Fig. 4:**
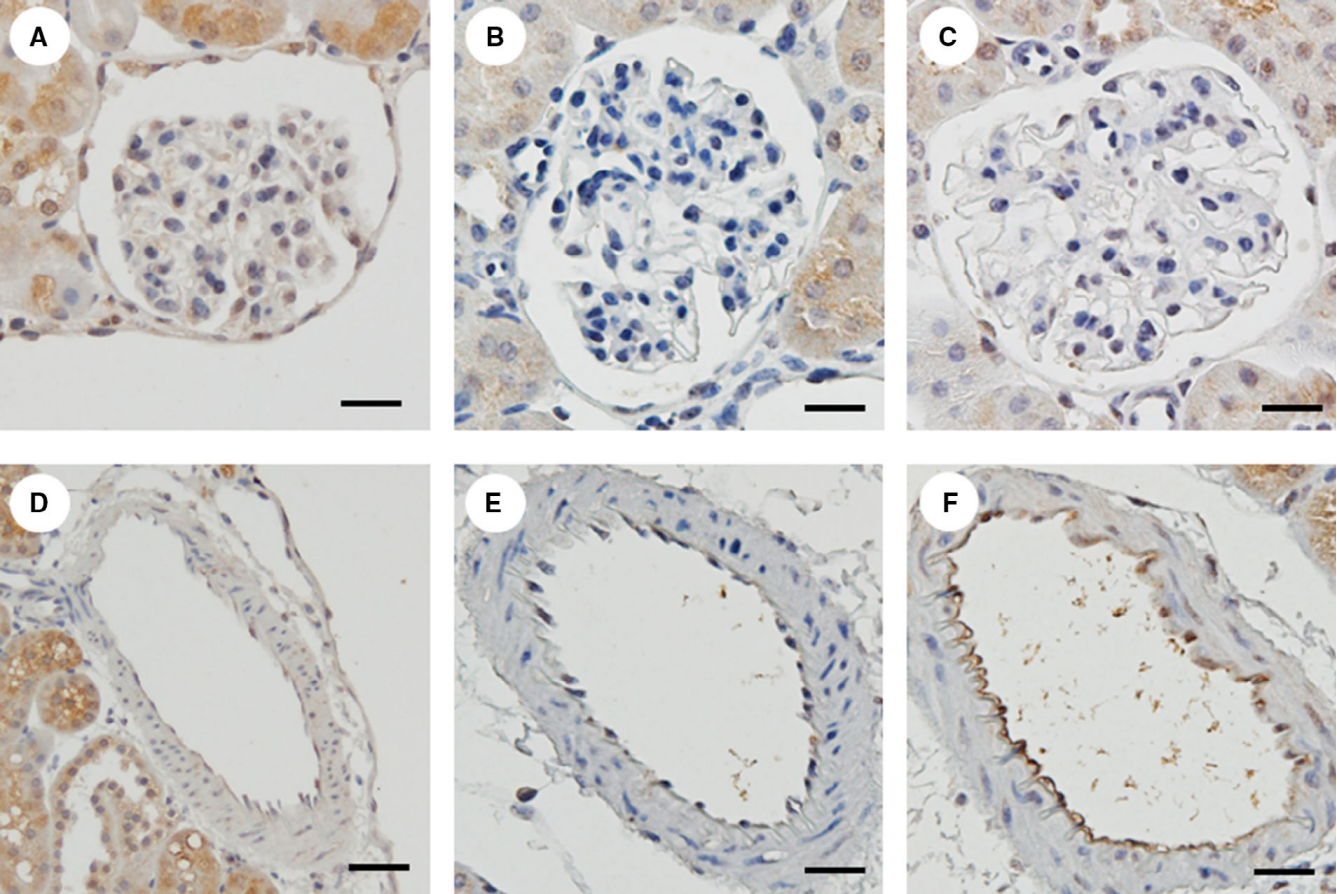
The immunohistochemical staining for eNOS. The levels of DAB in the glomerular capillaries and renal microvascular endothelial cells seemed to be higher in the Tx group compared with the DKD group. Representative kidney sections of immunohistochemical staining for eNOS in the Control (A), DKD (B), and Tx groups (C). Representative microvascular sections of immunohistochemical staining for eNOS in the Control (D), DKD (E), and Tx groups (F). The length of each scale bar shows 20 µm.

**Fig. 5:**
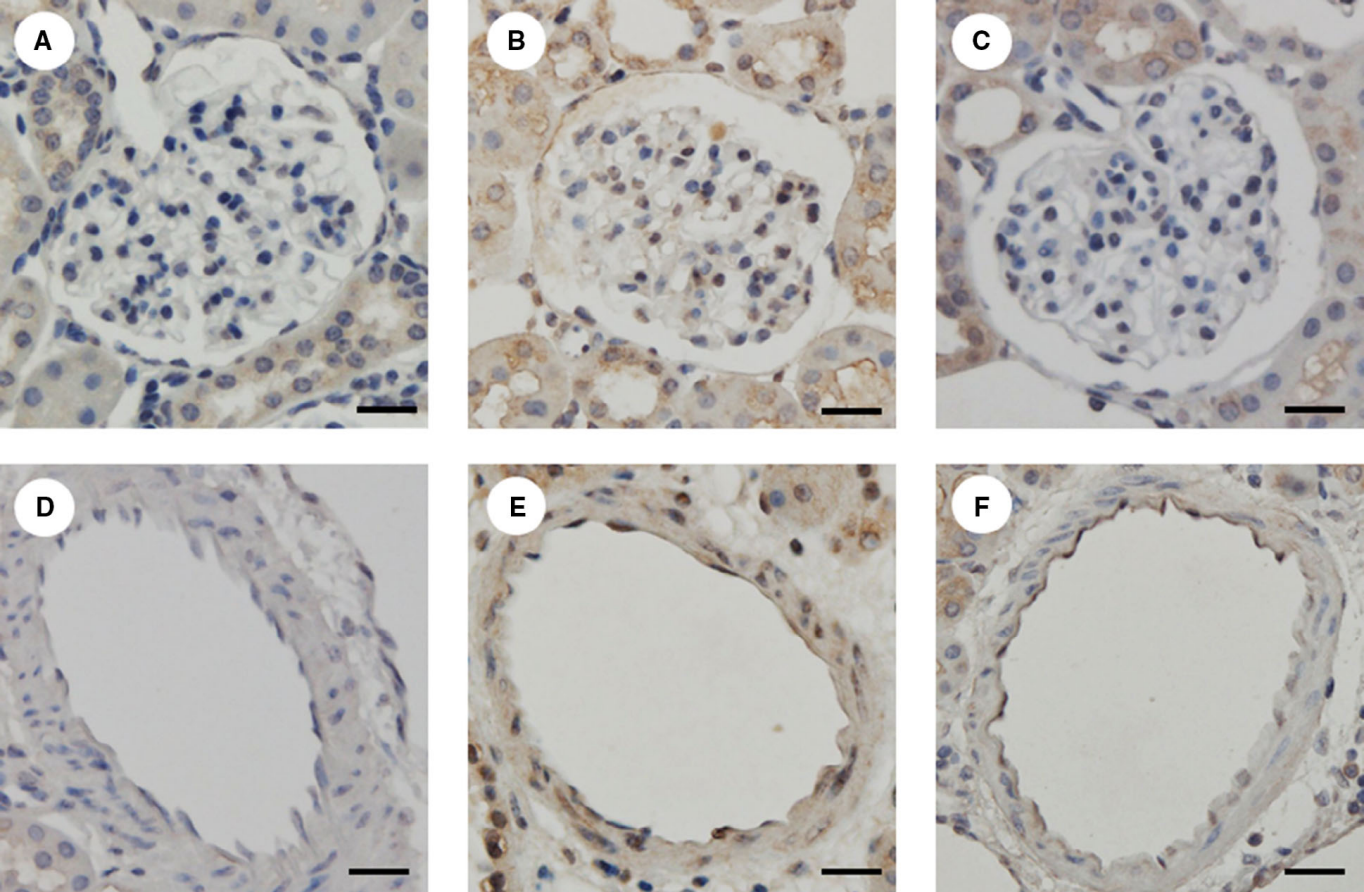
The immunohistochemical staining for 8‐OHdG. The levels of DAB in the cell nuclei of mesangial cells and renal microvascular smooth muscle cells seemed to be lower in the Tx group compared with the DKD group. Representative kidney sections of immunohistochemical staining for 8‐OHdG in the Control (A), DKD (B), and Tx groups (C). Representative microvascular sections of immunohistochemical staining for 8‐OHdG in the Control (D), DKD (E), and Tx groups (F). The length of each scale bar shows 20 µm.

Immunohistochemical staining for eNOS (Fig. 4) and 8‐OHdG (Fig. 5) was mainly localized in the glomeruli and microvasculature. Accumulations of eNOS at the glomerular capillaries and renal microvascular endothelial cells in the Tx group seemed to be stained more intensely than those in the DKD group. Additionally, the expression levels of 8‐OHdG in the nuclei of mesangial cells and renal microvascular smooth muscle cells appeared to be lower in the Tx group than in the DKD group.

### Urine nitrate and nitrite levels are not altered after Asp supplementation in the DKD mice

**Fig. 6:**
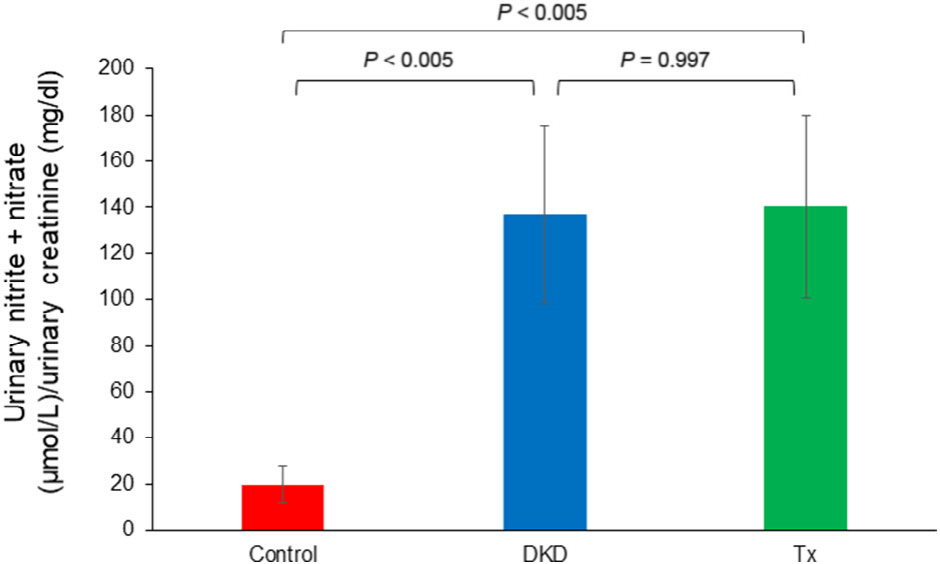
The level of urinary nitrates + nitrites. Urinary levels of nitrates + nitrites in the DKD group at 15 weeks of age were significantly higher than those in the Control group. Those levels did not differ between the DKD and Tx groups. The *Y*‐axis shows the concentration of urinary nitrates + nitrites (µmol·L^−1^) divided by urinary creatinine values (mg·dL^−1^). The error bars represent SD.

As shown in Fig. 6, urinary levels of nitrates + nitrites in the DKD group at 15 weeks of age were significantly higher than those in the Control group. On the other hand, those levels did not differ between the DKD and Tx groups.

## Discussion

The present study demonstrated that circulating Asp levels in diabetic mice were decreased, and Asp supplementation might be useful to prevent the progression of DKD via amelioration of endothelial function.

Asp, one of the nonessential amino acids, is required for the metabolic pathway from Citrulline to Arg. Arg is known as a substrate for NO. The NOS is responsible for the synthesis of NO, a reaction that occurs in all tissues. There are three kinds of NOS families that are differentially localized: nNOS is mainly present in neural cells, iNOS in macrophages, and eNOS in endothelial cells. The three enzymes share a common mechanism for synthesizing NO from Arg. When NO is made synthetically from Arg, Citrulline is also released as a byproduct of NO [12]. Some researchers have already reported results of basic studies on the supplementation with Arg and/or Citrulline for diabetes or DKD. Hayashi *et al*. [15] reported that the administration of Arg or Citrulline resulted in a significant recovery of the decreased nitrite level of the culture medium obtained from endothelial cells from the human umbilical vein cultured under high‐glucose condition. When Arg and Citrulline were given together, the recovery of nitrite production was more marked than when Arg alone was given [15]. Romero *et al*. [23] reported that supplementation with Citrulline reduced albuminuria, fibrosis, and kidney hypertrophy in streptozotocin‐induced diabetic mice. In contrast, supplementation with Citrulline, but not Arg, improved glomerular hyperfiltration, thereby markedly decreasing proteinuria in diabetic rats [14]. One may think that Arg supplementation is more effective to promote NO synthesis compared to Citrulline supplementation. However, oral Arg supplementation is largely ineffective due to gastrointestinal and hepatic extraction of Arg. Alternatively, oral Citrulline supplementation consistently increases the bioavailability of Arg and NO in plasma and tissue [24]. Most of the orally administered Citrulline is removed from circulation by the kidneys, and it is efficiently converted into Arg [25]. In our CE‐MS analysis, it appears that in KK‐Akita mice, the Citrulline levels absorbed were appropriate. However, circulating Arg levels in KK‐Akita mice were not different from those in KK mice. Additionally, eNOS expression in the glomeruli and microvessels of KK‐Akita mice seemed to be lower than those in KK mice. These results could be attributable to a lack of Asp, which is essential for the metabolism of Citrulline to Arg. Generally, plasmatic Arg concentrations are decreased in both humans and animals with diabetes [14, 26, 27]. This might be partially explained by the increased plasma arginase activity in diabetes [28, 29, 30]. As such, the bioavailability of Arg and NO production is reduced in the diabetic state. In fact, studies of arginase inhibition in patients with diabetes or diabetic rats have shown an improvement of microvascular endothelial function [13, 31, 32]. Based on these results, we guessed that Asp was consumed to sustain the bioavailability of Arg in KK‐Akita mice. Interestingly, circulating Asp levels were also significantly decreased in other diabetic models such as type 2 diabetic KK‐A^y^ mice compared with those of KK mice (data not shown).

The glomerular filtration barrier is composed of glomerular endothelial cells, GBM, and glomerular epithelial cells [33]. Glomerular endothelial cells are covered by ESL. ESL is mainly composed of the negatively charged heparan sulfate proteoglycan as well as albumin. It is known that increased oxidative stress induces glomerular ESL deterioration in part through increased heparanase levels, resulting in exacerbation of glomerular permselectivity and development of albuminuria [34, 35, 36]. In the present study, supplementation with Asp improved renal oxidative stress and structures of ESL in diabetic mice. The reduction in the rate of foot process effacement with the amelioration of ESL might be related to the reduction in GBM thickening, because injured podocytes contribute to the perturbation of the balance between the synthetic and degradative pathways of GBM [37]. However, the circulating concentrations of Asp and Citrulline did not change between the DKD and Tx groups. It is likely that supplemented Arg is consumed in the synthesis of NO, and citrulline is recreated as a byproduct of NO. Indeed, the pattern of changes in the ratio of Arg to citrulline, which reflects the relative activity of systemic NOS [32], among the three groups was similar to that observed for urinary nitrite + nitrate levels, indicating that NO production in KK‐Akita mice was probably higher than that in KK mice.

A limitation of the present study is that we were unable to determine direct NO production because the half‐life of NO is very short. Therefore, we measured the levels of urinary nitrite + nitrate as a stable oxidation products of NO (i.e., as indexes of NO production), although those levels seem to reflect not only local kidney but also systemic levels [38, 39]. Systemic NO production is shifted toward an increase during the early phases of DKD and leads to glomerular hyperfiltration, and eventually albuminuria [39, 40, 41, 42]. In the present study, the levels of urinary nitrite + nitrate in KK‐Akita mice were significantly higher than those in KK mice and did not change after Asp supplementation. These results are likely inherent to the diabetic condition. The levels of systemic NO production were reported to be associated positively with serum glucose levels [39, 43, 44]. We guess that decreased 8‐OHdG expression in the kidneys of mice in the Tx group resulted from the upregulation of glomerular endothelial NO production. Another limitation of this study is that KK‐Akita mice are unique diabetic model mice. They were generated by backcrossing female KK mice with male C57BL/6‐Akita mice, which are nonobese hypoinsulinemic diabetic mice. Thus, the results of the present study may not apply to other diabetic model mice.

In conclusion, we demonstrated that the Asp treatment induced the reduction of oxidative stress in the kidneys, amelioration of glomerular ESL, and suppression of albuminuria in KK‐Akita mice. It is possible that low serum concentration of Asp has some impact on DKD progression.

## Conflict of interest

The authors declare no conflict of interest.

## Author contributions

SI, TG, and YS conceived and designed the project; SI, ZL, EA, and TK acquired the data; SI, TG, and MM analyzed and interpreted the data; SI and TG wrote the paper.

## References

[1] Rossing K , Mischak H , Rossing P , Schanstra JP , Wiseman A and Maahs DM (2008) The urinary proteome in diabetes and diabetes‐associated complications: new ways to assess disease progression and evaluate therapy. Proteomics Clin Appl 2, 997–1007. 21136900 10.1002/prca.200780166

[2] Bjornstad P , Cherney DZ , Maahs DM and Nadeau KJ (2016) Diabetic kidney disease in adolescents with type 2 diabetes: new insights and potential therapies. Curr Diab Rep 16, 11. 26803647 10.1007/s11892-015-0708-0 PMC5841446

[3] Mottl AK , Kwon KS , Mauer M , Mayer‐Davis EJ , Hogan SL and Kshirsagar AV (2013) Normoalbuminuric diabetic kidney disease in the U.S. population. J Diabetes Complications 27, 123–127. 23182925 10.1016/j.jdiacomp.2012.09.010 PMC4594950

[4] Keri KC , Samji NS and Blumenthal S (2018) Diabetic nephropathy: newer therapeutic perspectives. J Community Hosp Intern Med Perspect 8, 200–207. 30181826 10.1080/20009666.2018.1500423 PMC6116149

[5] Fouli GE and Gnudi L (2019) The future: experimental therapies for renal disease in diabetes. Nephron 143, 3–7. 30257247 10.1159/000492825

[6] Perez‐Morales RE , Del Pino MD , Valdivielso JM , Ortiz A , Mora‐Fernandez C and Navarro‐Gonzalez JF (2019) Inflammation in diabetic kidney disease. Nephron 143, 12–16. 30273931 10.1159/000493278

[7] Nicholson JK (2006) Global systems biology, personalized medicine and molecular epidemiology. Mol Syst Biol 2, 52. 17016518 10.1038/msb4100095 PMC1682018

[8] Guasch‐Ferre M , Hruby A , Toledo E , Clish CB , Martinez‐Gonzalez MA , Salas‐Salvado J and Hu FB (2016) Metabolomics in prediabetes and diabetes: a systematic review and meta‐analysis. Diabetes Care 39, 833–846. 27208380 10.2337/dc15-2251 PMC4839172

[9] Decramer S , Wittke S , Mischak H , Zurbig P , Walden M , Bouissou F , Bascands JL and Schanstra JP (2006) Predicting the clinical outcome of congenital unilateral ureteropelvic junction obstruction in newborn by urinary proteome analysis. Nat Med 12, 398–400. 16550189 10.1038/nm1384

[10] Rossing K , Mischak H , Parving HH , Christensen PK , Walden M , Hillmann M and Kaiser T (2005) Impact of diabetic nephropathy and angiotensin II receptor blockade on urinary polypeptide patterns. Kidney Int 68, 193–205. 15954909 10.1111/j.1523-1755.2005.00394.x

[11] Fujita H , Fujishima H , Chida S , Takahashi K , Qi Z , Kanetsuna Y , Breyer MD , Harris RC , Yamada Y and Takahashi T (2009) Reduction of renal superoxide dismutase in progressive diabetic nephropathy. J Am Soc Nephrol 20, 1303–1313. 19470681 10.1681/ASN.2008080844 PMC2689908

[12] Curis E , Nicolis I , Moinard C , Osowska S , Zerrouk N , Benazeth S and Cynober L (2005) Almost all about citrulline in mammals. Amino Acids 29, 177–205. 16082501 10.1007/s00726-005-0235-4

[13] Gronros J , Jung C , Lundberg JO , Cerrato R , Ostenson CG and Pernow J (2011) Arginase inhibition restores in vivo coronary microvascular function in type 2 diabetic rats. Am J Physiol Heart Circ Physiol 300, H1174–H1181. 21297024 10.1152/ajpheart.00560.2010

[14] Persson P , Fasching A , Teerlink T , Hansell P and Palm F (2014) L‐Citrulline, but not L‐arginine, prevents diabetes mellitus‐induced glomerular hyperfiltration and proteinuria in rat. Hypertension 64, 323–329. 24866144 10.1161/HYPERTENSIONAHA.114.03519

[15] Hayashi T , Matsui‐Hirai H , Miyazaki‐Akita A , Fukatsu A , Funami J , Ding QF , Kamalanathan S , Hattori Y , Ignarro LJ and Iguchi A (2006) Endothelial cellular senescence is inhibited by nitric oxide: implications in atherosclerosis associated with menopause and diabetes. Proc Natl Acad Sci USA 103, 17018–17023. 17075048 10.1073/pnas.0607873103 PMC1629003

[16] Wang J , Takeuchi T , Tanaka S , Kubo SK , Kayo T , Lu D , Takata K , Koizumi A and Izumi T (1999) A mutation in the insulin 2 gene induces diabetes with severe pancreatic beta‐cell dysfunction in the Mody mouse. J Clin Invest 103, 27–37. 9884331 10.1172/JCI4431 PMC407861

[17] Lancha AH Jr , Poortmans JR and Pereira LO (2009) The effect of 5 days of aspartate and asparagine supplementation on glucose transport activity in rat muscle. Cell Biochem Funct 27, 552–557. 19821260 10.1002/cbf.1606

[18] Kasahara M , Mukoyama M , Sugawara A , Makino H , Suganami T , Ogawa Y , Nakagawa M , Yahata K , Goto M , Ishibashi R *et al* (2000) Ameliorated glomerular injury in mice overexpressing brain natriuretic peptide with renal ablation. J Am Soc Nephrol 11, 1691–1701. 10966494 10.1681/ASN.V1191691

[19] Kataoka H , Ohara M , Honda K , Mochizuki T and Nitta K (2011) Maximal glomerular diameter as a 10‐year prognostic indicator for IgA nephropathy. Nephrol Dial Transplant 26, 3937–3943. 21427079 10.1093/ndt/gfr139

[20] Ishizaka M , Gohda T , Takagi M , Omote K , Sonoda Y , Oliva Trejo JA , Asao R , Hidaka T , Asanuma K , Horikoshi S *et al* (2015) Podocyte‐specific deletion of Rac1 leads to aggravation of renal injury in STZ‐induced diabetic mice. Biochem Biophys Res Commun 467, 549–555. 26435502 10.1016/j.bbrc.2015.09.158

[21] Kakani S , Yardeni T , Poling J , Ciccone C , Niethamer T , Klootwijk ED , Manoli I , Darvish D , Hoogstraten‐Miller S , Zerfas P *et al* (2012) The Gne M712T mouse as a model for human glomerulopathy. Am J Pathol 180, 1431–1440. 22322304 10.1016/j.ajpath.2011.12.023 PMC3349896

[22] Wang L , Liu S , Yang W , Yu H , Zhang L , Ma P , Wu P , Li X , Cho K , Xue S *et al* (2017) Plasma amino acid profile in patients with aortic dissection. Sci Rep. 7, 40146. 28071727 10.1038/srep40146 PMC5223271

[23] Romero MJ , Yao L , Sridhar S , Bhatta A , Dou H , Ramesh G , Brands MW , Pollock DM , Caldwell RB , Cederbaum SD *et al* (2013) l‐Citrulline protects from kidney damage in type 1 diabetic mice. Front Immunol 4, 480. 24400007 10.3389/fimmu.2013.00480 PMC3871963

[24] Allerton TD , Proctor DN , Stephens JM , Dugas TR , Spielmann G and Irving BA (2018) l‐Citrulline supplementation: impact on cardiometabolic health. Nutrients 10, E921. 30029482 10.3390/nu10070921 PMC6073798

[25] Windmueller HG and Spaeth AE (1981) Source and fate of circulating citrulline. Am J Physiol 241, E473–E480. 7325229 10.1152/ajpendo.1981.241.6.E473

[26] Palm F , Friederich M , Carlsson PO , Hansell P , Teerlink T and Liss P (2008) Reduced nitric oxide in diabetic kidneys due to increased hepatic arginine metabolism: implications for renomedullary oxygen availability. Am J Physiol Renal Physiol 294, F30–F37. 17942569 10.1152/ajprenal.00166.2007

[27] Saleem T , Dahpy M , Ezzat G , Abdelrahman G , Abdel‐Aziz E and Farghaly R (2019) The profile of plasma free amino acids in type 2 diabetes mellitus with insulin resistance: association with microalbuminuria and macroalbuminuria. Appl Biochem Biotechnol 188, 854–867. 30706418 10.1007/s12010-019-02956-9

[28] Elms SC , Toque HA , Rojas M , Xu Z , Caldwell RW and Caldwell RB (2013) The role of arginase I in diabetes‐induced retinal vascular dysfunction in mouse and rat models of diabetes. Diabetologia 56, 654–662. 23232640 10.1007/s00125-012-2789-5 PMC3565067

[29] Romero MJ , Iddings JA , Platt DH , Ali MI , Cederbaum SD , Stepp DW , Caldwell RB and Caldwell RW (2012) Diabetes‐induced vascular dysfunction involves arginase I. Am J Physiol Heart Circ Physiol 302, H159–H166. 22058149 10.1152/ajpheart.00774.2011 PMC3334242

[30] Romero MJ , Platt DH , Tawfik HE , Labazi M , El‐Remessy AB , Bartoli M , Caldwell RB and Caldwell RW (2008) Diabetes‐induced coronary vascular dysfunction involves increased arginase activity. Circ Res 102, 95–102. 17967788 10.1161/CIRCRESAHA.107.155028 PMC2822539

[31] Shemyakin A , Kovamees O , Rafnsson A , Bohm F , Svenarud P , Settergren M , Jung C and Pernow J (2012) Arginase inhibition improves endothelial function in patients with coronary artery disease and type 2 diabetes mellitus. Circulation 126, 2943–2950. 23183942 10.1161/CIRCULATIONAHA.112.140335

[32] Kovamees O , Shemyakin A , Checa A , Wheelock CE , Lundberg JO , Ostenson CG and Pernow J (2016) Arginase inhibition improves microvascular endothelial function in patients with type 2 diabetes mellitus. J Clin Endocrinol Metab 101, 3952–3958. 27399350 10.1210/jc.2016-2007

[33] Haraldsson B , Nystrom J and Deen WM (2008) Properties of the glomerular barrier and mechanisms of proteinuria. Physiol Rev 88, 451–487. 18391170 10.1152/physrev.00055.2006

[34] Kuwabara A , Satoh M , Tomita N , Sasaki T and Kashihara N (2010) Deterioration of glomerular endothelial surface layer induced by oxidative stress is implicated in altered permeability of macromolecules in Zucker fatty rats. Diabetologia 53, 2056–2065. 20526760 10.1007/s00125-010-1810-0 PMC2910881

[35] Singh A , Satchell SC , Neal CR , McKenzie EA , Tooke JE and Mathieson PW (2007) Glomerular endothelial glycocalyx constitutes a barrier to protein permeability. J Am Soc Nephrol 18, 2885–2893. 17942961 10.1681/ASN.2007010119

[36] van den Berg BM , Vink H and Spaan JA (2003) The endothelial glycocalyx protects against myocardial edema. Circ Res 92, 592–594. 12637366 10.1161/01.RES.0000065917.53950.75

[37] Marshall CB (2016) Rethinking glomerular basement membrane thickening in diabetic nephropathy: adaptive or pathogenic? Am J Physiol Renal Physiol 311, F831–F843. 27582102 10.1152/ajprenal.00313.2016 PMC6121820

[38] Bank N and Aynedjian HS (1993) Role of EDRF (nitric oxide) in diabetic renal hyperfiltration. Kidney Int 43, 1306–1312. 8315943 10.1038/ki.1993.183

[39] Apakkan Aksun S , Ozmen B , Ozmen D , Parildar Z , Senol B , Habif S , Mutaf I , Turgan N and Bayindir O (2003) Serum and urinary nitric oxide in type 2 diabetes with or without microalbuminuria: relation to glomerular hyperfiltration. J Diabetes Complications 17, 343–348. 14583179 10.1016/s1056-8727(02)00196-4

[40] Dellamea BS , Leitao CB , Friedman R and Canani LH (2014) Nitric oxide system and diabetic nephropathy. Diabetol Metab Syndr 6, 17. 24520999 10.1186/1758-5996-6-17 PMC3928920

[41] Ishii N , Patel KP , Lane PH , Taylor T , Bian K , Murad F , Pollock JS and Carmines PK (2001) Nitric oxide synthesis and oxidative stress in the renal cortex of rats with diabetes mellitus. J Am Soc Nephrol 12, 1630–1639. 11461935 10.1681/ASN.V1281630

[42] Komers R and Anderson S (2003) Paradoxes of nitric oxide in the diabetic kidney. Am J Physiol Renal Physiol 284, F1121–F1137. 12736164 10.1152/ajprenal.00265.2002

[43] Adela R , Nethi SK , Bagul PK , Barui AK , Mattapally S , Kuncha M , Patra CR , Reddy PN and Banerjee SK (2015) Hyperglycaemia enhances nitric oxide production in diabetes: a study from South Indian patients. PLoS ONE 10, e0125270. 25894234 10.1371/journal.pone.0125270 PMC4403926

[44] Verma S , Alam R , Ahmad I , Singla D , Ali K and Hussain ME (2018) Effect of glycemic control and disease duration on cardiac autonomic function and oxidative stress in type 2 diabetes mellitus. J Diabetes Metab Disord 17, 149–158. 30918849 10.1007/s40200-018-0354-6 PMC6405381

